# Sensitive Non-Enzymatic Glucose Electrochemical Sensor Based on Electrochemically Synthesized PANI/Bimetallic Oxide Composite

**DOI:** 10.3390/polym14153047

**Published:** 2022-07-27

**Authors:** Anish Khan, Aftab Aslam Parwaz Khan, Hadi M. Marwani, Maha Moteb Alotaibi, Abdullah M. Asiri, Ayyar Manikandan, Suchart Siengchin, Sanjay Mavinkere Rangappa

**Affiliations:** 1 Center of Excellence for Advanced Materials Research, King Abdulaziz University, Jeddah 21589, Saudi Arabia; draapk@gmail.com (A.A.P.K.); hmarwani@kau.edu.sa (H.M.M.); aasiri2@gmail.com (A.M.A.); 2 Chemistry Department, Faculty of Science, King Abdulaziz University, Jeddah 21589, Saudi Arabia; mmsalotaibi@kau.edu.sa; 3 Department of Chemistry, Bharath Institute of Higher Education and Research (BIHER), Bharath University, Chennai 600073, India; manikandana.che@bharathuniv.ac.in; 4 Natural Composites Research Group Lab, Department of Materials and Production Engineering, The Sirindhorn International Thai-German Graduate School of Engineering (TGGS), King Mongkut’s University of Technology North Bangkok (KMUTNB), Bangkok 10800, Thailand; suchart.s.pe@tggs-bangkok.org (S.S.); mavinkere.r.s@op.kmutnb.ac.th (S.M.R.)

**Keywords:** electrochemical sensor, glucose sensor, PANI-MnBaO_2_, conducting polymer composite, cyclic voltammetry, linear sweep voltammetry

## Abstract

The development of a sensitive glucose monitoring system is highly important to protect human lives as high blood-glucose level-related diseases continue to rise globally. In this study, a glucose sensor based on polyaniline-bimetallic oxide (PANI-MnBaO_2_) was reported. PANI-MnBaO_2_ was electrochemically synthesized on the glassy carbon electrode (GCE) surface. The as-prepared PANI-MnBaO_2_ was characterized by field emission scanning electron microscopy, Fourier transform infrared spectroscopy, energy dispersive X-ray spectroscopy, cyclic voltammetry, and electrochemical impedance spectroscopy. Glucose sensing on PANI-MnBaO_2_ is based on the electrocatalytic oxidation of glucose to the glucolactone, which gives oxidation current. The oxidation potential for glucose was 0.83 V, with a limit of detection of 0.06 µM in the linear and in the concentration range of 0.05 µM–1.6 mM. The generated current densities displayed excellent stability in terms of repeatability and reproducibility with fast response. The development of a sensitive glucose sensor as obtained in the current study would ensure human health safety and protection through timely and accurate glucose detection and monitoring.

## 1. Introduction

Cases of diabetes have been increasing rapidly in the last decades globally, and the International Diabetic Federation has projected that by 2035, cases of diabetes will reach 600 million globally [1]. High blood-glucose levels in the human system are the major cause of diabetic cases. The glucose level in the human system exceeding 6.5 mM is a signal of the onset of diabetes [2,3]. In addition, high glucose levels in the human body could lead to malfunction or damage to the vital organs such as the heart, eyes, kidneys, tissues, and blood vessels [4,5,6]. Therefore, there is a great need for accurate and timely detection of glucose to protect and save human lives. In the past and till now, several analytical techniques have been used to determine glucose level/concentration. These techniques include colometric, spectroscopic and electrochemical methods. Electrochemical-based sensing of glucose is very attractive among the techniques because it provides low-cost, accurate detection in a short time and is a simple process. Electrochemical glucose sensing can be categorized into enzymatic and non-enzymatic detection. Non-enzymatic detection offers much more affordable, selectivity, sensitivity, and performance reproducibility [7,8,9].

Previously, researchers have explored the use of nanomaterials based on metal oxides, metal phosphides, metal sulfides, double-layered hydroxides, etc., for non-enzymatic glucose detection. Similarly, the use of conducting polymer-based materials such as PANI, polypyrrole composites have been reported. For instance, PANI microtube-modified electrode exhibited glucose detection in the linear range of 0.004–0.8 mM and a limit of detection (LOD) of 0.8 µM [10]. Wenwei et al., tried to increase the linear range of glucose using PANI by incorporating TiO_2_ into PANi film. The obtained linear range stretched from 0.02–6 mM and LOD of 6.31 µM [11]. Similarly, the incorporation of NiFe nanoparticles into PANI yielded LOD of 0.5 µM in the linear range of 10 µM–1 mM [12]. Recently, Liu et al., synthesized PANI fibers with CuO nanoparticle fillers for the detection of glucose at LOD of 0.11 µM [13]. In a recent study by Varghese et al., PANI doped Ag nanoparticle was synthesized and applied as a non-enzymatic glucose sensor. Their procedure achieved a detection limit of 1.3 µM in the linear range of 100 µM to 10 mM [14]. However, despite several studies on glucose electrochemical detection, there is a need for improvement of sensor performance in terms of selectivity, sensitivity, stability, and response time [14,15,16]. To address these challenges, this study reports the synthesis of PANI-MnBaO_2_ for glucose detection. Selection of conducting polymer such as PANI in composite with manganous and barium oxide is conceived because of their individual unique properties which include high electrical conductivity, high catalytic property, and chemical stability. PANI has excellent electrochemical properties, biocompatibility, and ease of synthesis which prompt its use as an electrode surfaces modifier [17]. It also has conjugated pi-electron in the backbone of its carbon chain. This conjugated electron affords it high electron mobility which accounts for its high chemical stability, and electrical and catalytic properties. In addition, manganous oxide (MnO) is a transition metal oxide with high catalytic properties as a result of partially filled d-orbital in Mn., and also has good electrical conductivity [18]. Likewise, barium oxide (BaO) is a stable metal oxide with good chemical stability [19]. It is thought that composting these materials would result in a highly effective catalyst and a sensing material that could be suitable for non-enzymatic oxidation of glucose as a means of its detection.

Consequently, the aim of the current study is to develop sensitive and reliable electrochemical sensors for glucose determination using affordable and readily available materials. To the best of the authors’ knowledge, PANI-MnBaO_2_ has never been synthesized before and has never been applied for glucose sensing. This makes this study original, important, and a major contribution to glucose sensing technology. 

## 2. Materials and Methods

### 2.1. Reagents

The reagents used for this study were used as purchased without purification except for aniline, which was distilled before use. The reagents used include aniline hydrochloride, manganese II sulfate, barium nitrate, potassium chloride, potassium nitrate, potassium dihydrogen phosphate, dipotassium hydrogen phosphate, glucose, fructose, uric acid, ascorbic acid, nitrite, zinc sulfate, copper sulfate, nitrite, human serum albumin and deionized water. All the reagents used are analytical grades and were sourced from Sigma-Aldrich, St. Louis, MO, USA.

### 2.2. Apparatus

The following apparatus was used for this study: field emission scanning electron microscopy (FESEM) (JEOL JSAM 6300, Jeol, Tokyo, Japan), energy dispersive X-ray spectrometer (XEDS) (X-Max Oxford, Oxford Instruments, Abingdon, UK), Fourier transform Infrared spectrometer (Thermo Scientific, Waltham, MA, USA powered by Vision software). Others include an electrochemical workstation by Autolab Model AUT85887 (Utrecht, The Netherlands), a three-electrode system made up of a reference electrode (Ag/AgCl–3M (in KCl)), a working electrode made of glassy carbon electrode (bare or coated with PANI-MnBaO_2_ and counter electrode (Pt wire–3 mm diameter).

### 2.3. Experimental

#### 2.3.1. Synthesis of PANI

PANI was synthesized by the electrochemical method, specifically, by the chronoamperometric method. The choice of the chronoamperometric method was informed because of its advantages such as ease of formation of controllable film thickness as well as fast and rapid synthesis [20,21]. Before the synthesis, the electrochemical cell was saturated with nitrogen gas to remove dissolved oxygen, or any other gaseous bubbles and this process was repeated in every electrochemical measurement. In order to ascertain the oxidation potential of aniline, the potentiodynamic method (cyclic voltammetry) was employed. A potentiostatic voltage of 1.0 V (gotten from the cyclic voltammetric method) at room temperature was applied to the electrochemical cell containing 0.1 M H_2_SO_4_ and 0.5 M aniline using a scan rate of 75 mV/s. The formation of PANI was monitored closely as the synthesis proceeded with special attention to the accompanying color change. After the deposition of PANI on the GCE surface, the modified GCE was gently washed with de-ionized water to remove unpolymerized aniline and oligomers.

#### 2.3.2. Deposition of MnO and BaO on the PANI Support

After the completion of the deposition of the polymerized aniline substrate, the modified electrode was cleaned thoroughly and air-dried. The as-prepared electrode was then immersed in a solution containing 0.1 M manganese sulfate and 0.1 M KNO_3_ (supporting electrolyte). The electrochemical setup was subjected to a potentiodynamic sweep from 0 V to–1.0 V at a scan rate of 75 mV/s for 10 cycles. The same procedure was reported for the deposition of Ba^2+^ in a solution containing 0.1 M Ba (NO_3_)_2_ and 0.1 M KNO_3_. The modified GCE was then immersed in a solution containing 0.1 M NaOH to convert the Mn^2+^ and Ba^2+^ to their corresponding oxides, MnO and BaO respectively. The modified GCE was later then washed thoroughly with de-ionized water to remove unadsorbed ions. The as-prepared PANI-MnBaO_2_ was used for the subsequent experiment.

#### 2.3.3. Characterization Technique

The structure and morphology of the synthesized PANI-MnBaO_2_ were investigated using field emission scanning electron microscopy. The functionalities in the synthesized PANI composite were investigated with FTIR in the spectrum range of 400–4000 cm^−1^ (FTIR-ATR). Moreover, the elemental analysis to identify elemental composition was carried out with x-ray energy dispersive spectroscopy (XEDS) fitted with FESEM. Electrochemical properties of the synthesized composite were assessed with Autolab potentiostat with the aid of cyclic voltammetry (CV) and electrochemical impedance spectroscopy (EIS). CV was conducted in the potential window of 0.1 V to 1.2 V, with a scan rate of 75 mV/s and an amplitude of 10 mV. EIS was carried out at a potential of 400 mV.

#### 2.3.4. Application for Glucose Sensing

Electrochemical sensing of glucose was conducted using cyclic voltammetry (CV) and linear sweep voltammetry (LSV) with the following applied parameters. CV: potential window: 0.1 to 1.2 V, scan rate of 75 mV/s, amplitude of 0.005 V; LSV: potential window of 0.1 to 1.2 V, scan rate of 75 mV/s, and amplitude of 5 mV.

## 3. Results and Discussion

### 3.1. Synthesis of PANI

**Figure 1:**
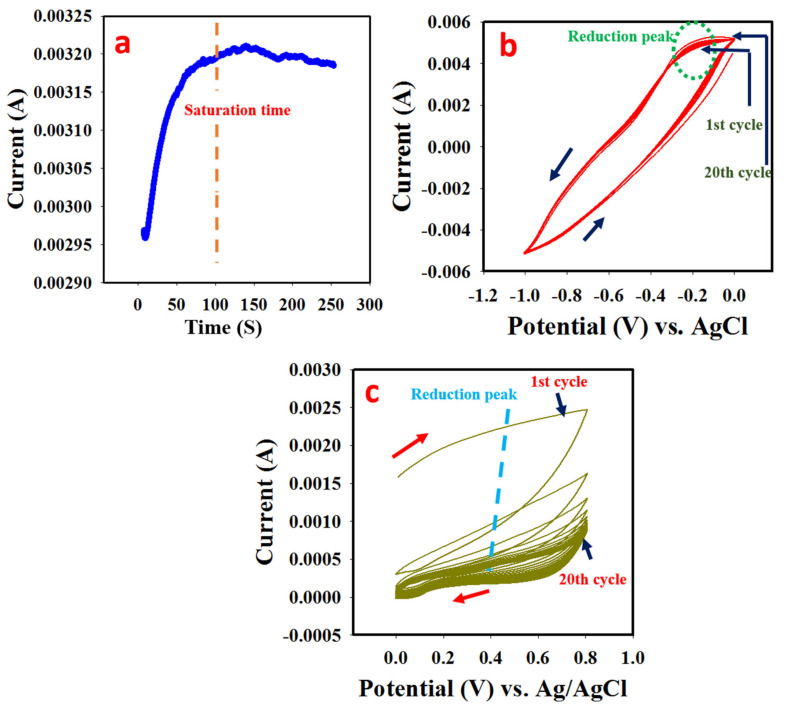
(**a**) Chronoamperogram obtained during synthesis of PANI at a constant potential of 1.0 V. (**b**) Cyclic voltammogram obtained during deposition of MnO on PANI coated GCE in a solution containing 0.1 M MnSO_4_/0.1 M KNO_3_ at a scan rate of 75 mV/s. (**c**) Cyclic voltammogram obtained during deposition of BaO on PANI−MnO coated GCE in a solution containing 0.1 M Ba(NO_3_)_2_/0.1 M KNO_3_ at a scan rate of 75 mV/s.

Before the electropolymerization, the color of aniline was white yellowish; but gradually changed to dark green (especially at the near working electrode region) upon passage of a constant potential of 1.0 V. This potential was selected based on obtained oxidation potential for aniline oxidation. A gradual rise in current was observed from 10 s which continued to rise till 120 s (Figure 1a). The increment in the current response (from about 10 s) was due to the gradual deposition of PANI which led to the observed increasing current. At about 120 s, the current started decreasing going forward, which could be attributed to the exhaustion of active sites on the glassy carbon electrode (Figure 1a). This phenomenon signified a successful synthesis of a conducting material (PANI).

### 3.2. Electrodeposition of MnO

The obtained cyclic voltammogram in 0.1 M manganese II sulfate is presented in Figure 1b. At 0.28 V, a peak could be observed which kept reducing with increasing cyclic sweep. At this potential, reduction of Mn^2+^ to Mn is suggested; The reduction peak decreased until the active sites on the substrate (PANI) were exhausted and therefore remained constant. It should be noted also that, the higher the cyclic sweep during the electro-deposition process, the higher the deposited materials and vice versa. The proposed equation of the reaction is presented in Equations (1)–(3) as adapted from the literature [22,23,24].

MnSO_4_ ⟶ Mn^2+^ + SO_4_^2−^ (1)

Mn ^2+^ + OH^−^ + e- ⟶ Mn (OH)_2_ (2)

Mn(OH)_2_ ⟶ MnO + H_2_O (3)

### 3.3. Electrodeposition of BaO

The as-prepared PANI-MnO was immersed in 0.1 M Ba (NO_3_)_2_/0.1 M KNO_3_ resulting in the obtained cyclic voltammogram shown in Figure 1c. At the potential of 0.36 V, a reduction process of Ba^2+^ was observed with the diminishing current response as the sweep increased. The deposited Ba (s) was converted to their respective oxides by a cyclic sweep in 0.1 M NaOH. The proposed equation of the reaction is presented in Equations (4) and (5) as adapted from the literature [22,23,24].

NO_3_^−^ + H_2_O + 2e^−^ ⟶ NO_2_^−^ + 2OH^−^ (4)

Ba ions precipitate with the hydroxyl anions and are spontaneously dehydrated into BaO.

Ba^2+^ + 2OH^−^ ⟶ Ba(OH)_2_ ⟶ BaO + H_2_O (5)

### 3.4. Morphological Studies

**Figure 2:**
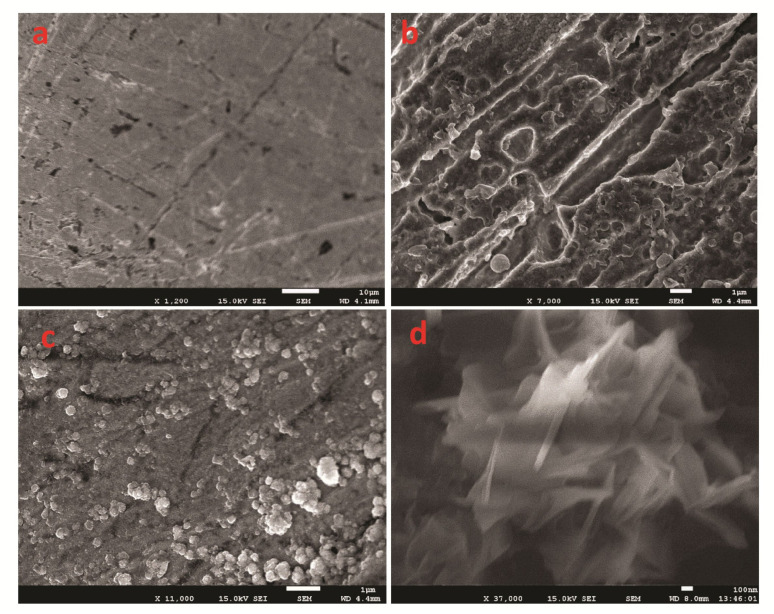
(**a**) Image of unmodified GCE (before electropolymerization). (**b**) Image of deposited PANI of GCE surface. (**c**) Image of PANI@MnBaO_2_ (low magnification). (**d**) Focus on MnBaO_2_ nanostructure (high magnification).

The structural and morphological image as captured by FESEM is presented in Figure 2. The image revealed a homogeneously coated surface. The deposit exhibits a non-porous amorphous look. However, upon deposition of MnBaO_2_, some agglomerated particles could be seen sparsely distributed on the PANI surface. The deposited particles (Figure 2c,d) suggest that particle growth predominated nucleation during the electro-synthesis of MnBaO_2_. At higher magnification, the deposited bimetallic oxide exhibited a crystalline sheet-like material with an average size of 15 nm.

First, the bare glassy carbon electrode as shown in Figure 2a revealed a plain and uncoated surface, slightly marred with little lining, which possibly arose from the electrode polishing. Upon deposition of PANI, a new semi-amorphous stricture could be observed. The crystallinity of the deposited material was enhanced upon doping with MnBaO_2_ fillers (Figure 2b–d).

### 3.5. Functionalities and Elemental Analysis

**Figure 3:**
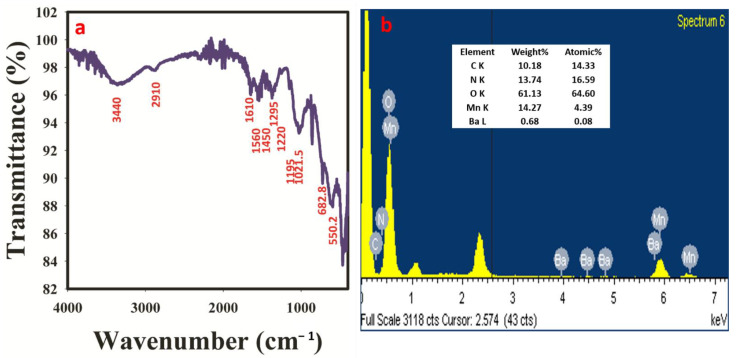
(**a**) FTIR spectrum of the synthesized PANI−MnBaO_2_ composite. (**b**) XEDS spectrum of the PANI−MnBaO_2_ composite.

The FTIR study was conducted to elucidate the functional groups in the synthesized materials. The obtained spectrum is shown in Figure 3a. Successful formation of PANI was confirmed with the following characteristic peaks peculiar to PANI. These characteristic peaks are 3400 cm^−1^ (N-H stretching vibration); 2910 cm^−1^ (C-H aromatic stretch); 1560 cm^−1^ (N = Q = N quinod stretch); 1290 cm^−1^ and 1230 cm^−1^ (C-N aromatic amine stretch); 1120 cm^−1^ and 990 cm^−1^ (protonated PANI—C-N^+^ stretch) [25,26,27]. Moreover, a distinct peak at 1600 cm^−1^ could be associated with Ba-O while the peaks at the fingerprint region (680 cm^−1^ and 550 cm^−1^) are typical of Mn-O vibration [28,29]. The obtained data from the FTIR spectrum confirms the successful synthesis of PANI and highly suggests successful MnO and BaO doping. This claim is further substantiated by the XEDS spectrum (Figure 3b) which revealed the presence of carbon, nitrogen, oxygen, manganese, and barium in the prepared material, which are the elemental features of the synthesized PANI-MnBaO_2_.

### 3.6. Material Characterization with Cyclic Voltammetry and EIS

**Figure 4:**
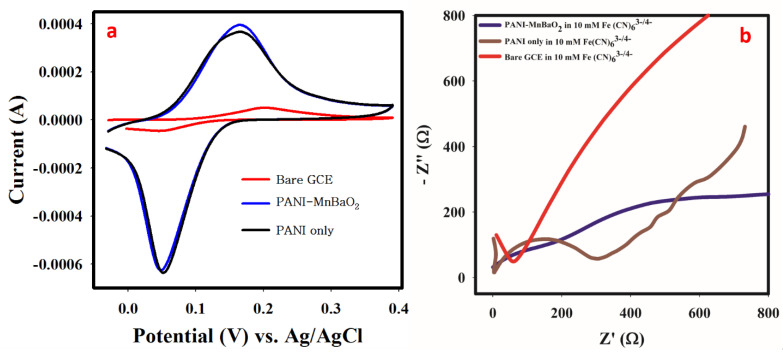
(**a**) Cyclic voltammogram obtained in 0.1 mM Fe (CN)_6_^3^−/^4^− at a scan rate of 75 mV/s. (**b**) EIS spectrum obtained in 10 mM Fe (CN)_6_^3^−/^4^−.

The electron mobility, which translates to the electrical conductivity of PANI−MnBaO_2_ was assessed with cyclic voltammetry using potassium ferricyanide as the supporting electrolyte. The obtained cyclic voltammogram obtained in 0.1 mM Fe(CN)_6_^3^−^/4^− is presented in Figure 4a. Information related to electron mobility and electrical conductivity can be inferred from the cyclic voltammogram. From Figure 4a, the oxidation peak current of PANI−MnBaO_2_ modified GCE was four times higher than that of bare GCE. Compared to PANI modified GCE only, improved electron mobility is observed with MnBaO_2_ doped PANI. In addition, anodic/cathodic peak separation potential is also smaller for the PANI−MnBaO_2_ modified GCE (101 mV), as compared to the bare GCE (149 mV). These phenomena are indications of improved electron mobility and electrical conductivity of the PANI−MnBaO_2_.

Electrochemical impedance spectroscopy was conducted purposely to investigate interfacial electron/charge transfer between the modified electrode surface and electrolyte. The Nyquist plot is useful to elucidate information on the resistance on the electrode surface which can be revealed by the charge transfer resistance (Rct) denoted by the semi-circle of the Nyquist plot [30,31]. A semi-circle or segment with a smaller radius or diameter has a better interfacial electron transfer compared to a bigger one. As given in Figure 4b and Table S2, PANI-MnBaO_2_ modified GCE displayed a reduced segment (Rct) (198 Ω) compared to the unmodified PANI (403 Ω) and bare GCE (1.47 kΩ) at high frequency. The reduced charge transfer resistance value of PANI-MnBaO_2_ indicates better electrochemical property than unmodified PANI and bare GCE. The equivalent circuit diagram for the reaction process with values of circuit parameters is given in ESI-S1. 

### 3.7. Electrochemical Response to Glucose

#### 3.7.1. Control Study and Optimization

**Figure 5:**
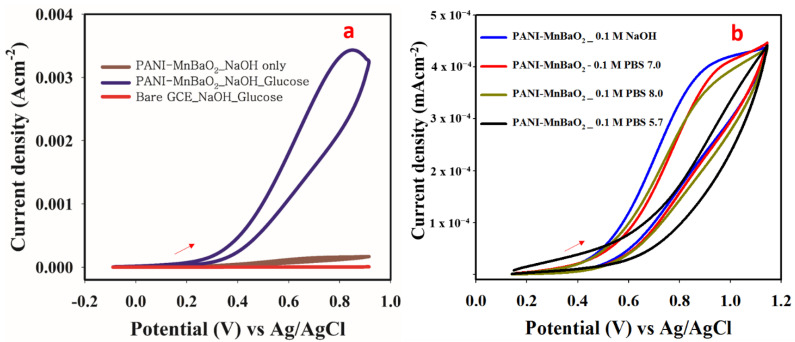
(**a**) Cyclic voltammogram obtained in a solution containing 20 mM glucose and 0.1 M NaOH.at a scan rate of 75 mV/s. (**b**) Cyclic voltammogram obtained in a solution containing 0.2 mM glucose and different pH/supporting electrolyte.

In order to establish a response from PANI-MnBaO_3_ towards glucose, a controlled study was used, involving bare GCE and modified GCE in glucose solution. PANI-MnBaO_2_ gives an oxidation peak at 0.83 V while bare GCE did not show any peak at this potential. Moreover, in the absence of glucose solution, PANI-MnBaO_3_ showed very little or no peak at 0.83 V. The obtained results indicate that PANI-MnBaO_2_ is responsible for the oxidation peak observed (Figure 5a). This suggests that PANI-MnBaO_2_ catalyzed the oxidation of glucose to gluconolactone due to its unique catalytic property. In order to optimize the glucose oxidation (basis of glucose sensing), supporting electrolyte optimization/effect was studied. From the result, the optimum supporting electrolyte for glucose oxidation was 0.1 M NaOH (pH 10) because it has the highest oxidation current at the lowest potential (Figure 5b). The result suggests that the oxidation of glucose was enhanced in the alkaline medium compared to the acidic medium.

#### 3.7.2. Calibration Curve, Scan Rate, and Response Stability

**Figure 6:**
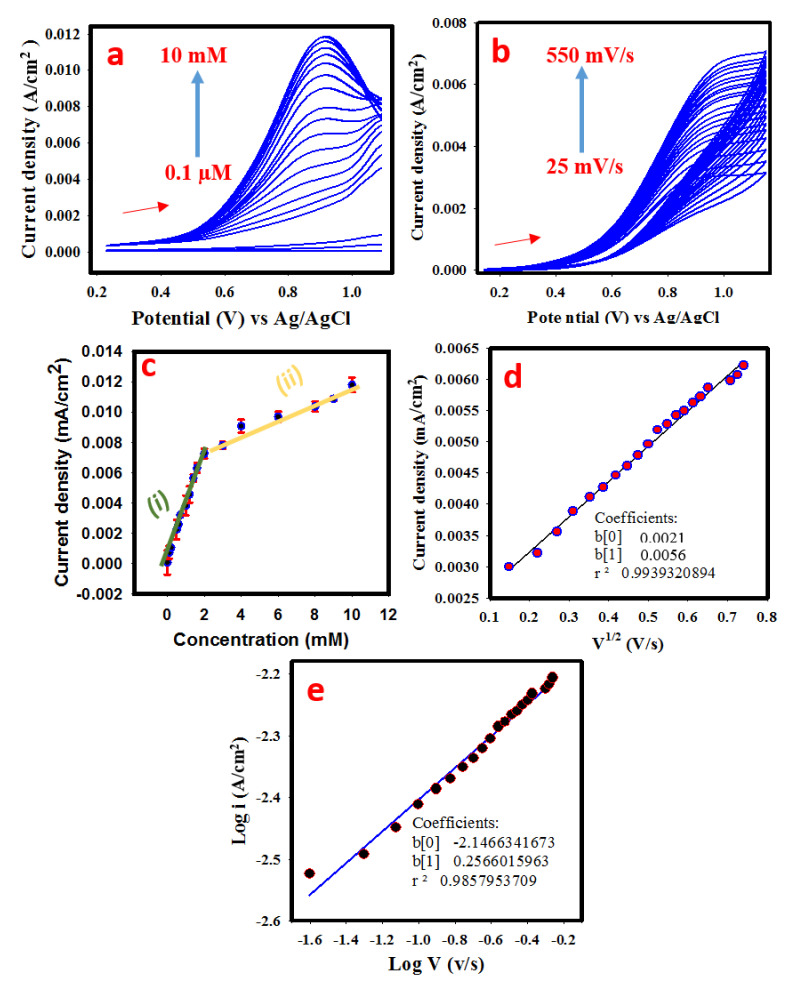
(**a**) Linear sweep voltammogram obtained at different glucose concentrations at the scan rate of 75 mV/s. (**b**) Cyclic voltammogram obtained in 2 mM glucose/0.1 M NaOH solution at the scan rate of 25 mV/s to 550 mV/s. (**c**) calibration plot with error bars. (**d**) Linear regression graph of oxidation current density against the square root of the scan rate. (**e**) Plot of the logarithm of oxidation current density against the logarithm of the scan rate.

The behavior of PANI-MnBaO_2_ towards glucose concentration is shown in Figure 6a. The linear sweep voltammogram showed that an increasing oxidation peak was observed from the addition of 0.1 µM glucose to 10 mM. The calibration curve (Figure 6b) with the equation of the graph (i) and (ii) has a good correlation and as presented in Equations (6) and (7).

i_p_ (ox) = 0.0004 × C (µM (±0.000109)) − 0.003 (±0.000104) (6)

i_p_ (ox) = 0.00672 × C (µM) (±0.00039)) + 0.005 (±0.00003) (7)

Figure 6c presents the cyclic voltammogram obtained at different scan rates (25 mV/s –550 mV/s). The effect of scan rate on the current response of an electrode material could give information about the diffusivity of the reaction on the electrode surface. The linear increase in scan rate and corresponding current portrays a diffusion-controlled reaction (as given in Figure 6d). Specifically, the slope of the plot of the logarithm of scan rate against the logarithm of peak current emphatically dictates whether a reaction is a fully diffusion-controlled or partial diffusion. As given in the literature [21], the slope of plot of logarithm of current against logarithm of scan rate < 0.4 denotes s fully diffusion-controlled reaction; 0.4–0.6 denotes a partial diffusion-controlled reaction; while >0.6 denotes adsorption-controlled reaction [31,32]. For this study, the plot of the square root of the scan rate is linearly proportional to the peak current which suggests a diffusion-controlled reaction.

From the calibration plot, the analytical performance of the electrode (sensor) was determined in terms of limit of detection (LOD), limit of quantification (LOQ), sensitivity, linear response, and dynamic range. The LOD, LOQ, and sensitivity were estimated using Equations (8)–(10), respectively.

(8) $$\mathrm{LOD}=\frac{3\times \;\mathrm{Sd}\;\left(\mathrm{standard}\;\mathrm{deviation}\;\mathrm{of}\;\mathrm{the}\;\mathrm{blank}\right)}{\mathrm{slope}\;\mathrm{of}\;\mathrm{the}\;\mathrm{calibration}}$$

(9) $$\mathrm{LOQ}=\frac{10\times \;\mathrm{Sd}\;\left(\mathrm{standard}\;\mathrm{deviation}\;\mathrm{of}\;\mathrm{the}\;\mathrm{blank}\right)}{\mathrm{slope}\;\mathrm{of}\;\mathrm{the}\;\mathrm{calibration}}$$

(10) $$\mathrm{Sensitivity}=\frac{\mathrm{Slope}\;\mathrm{of}\;\mathrm{the}\;\mathrm{calibration}}{\mathrm{GCE}\;\mathrm{surface}\;\mathrm{area}}$$

The values obtained for LOD, LOQ, and sensitivity are 0.06 µM, 0.199 µM, and 128 µAmM^−1^ cm^−2^ respectively. The linear response was recorded in the range of 0.05–1.6 mM while the full dynamic response was in the range of 0.05–10 mM.

**Figure 7:**
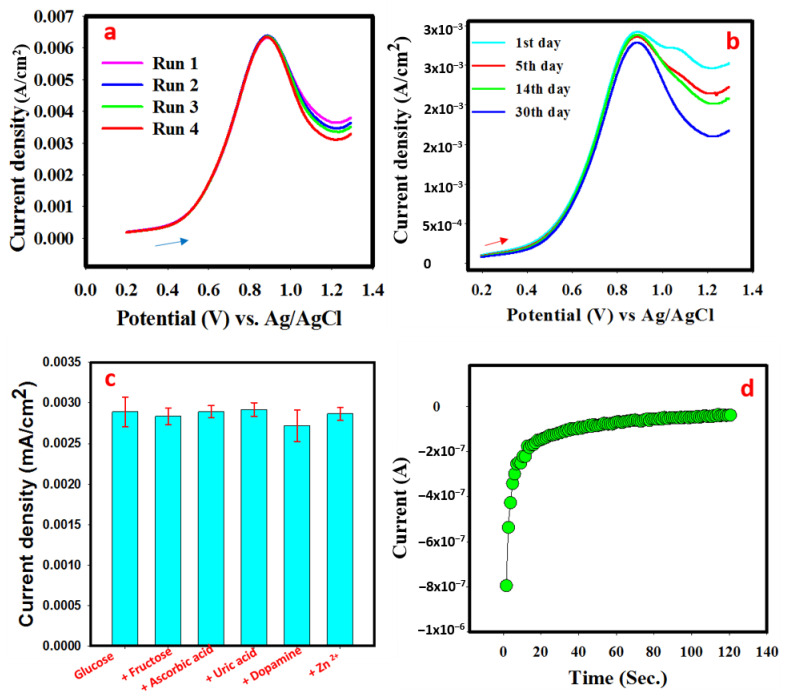
(**a**) Current density response of PANI-MnBaO_2_ in 4 mM glucose performed at five consecutive times. (**b**) LSV response of PANI-MnBaO_2_ in 2 mM glucose obtained over a period of 30 days. (**c**) Peak current density in 2 mM glucose (with and without likely interferents). (**d**) the obtained response time in 2 mM glucose solution.

In addition, the response of PANI-MnBaO_2_ modified GCE towards glucose sensing was assessed in terms of repeatability, reproducibility, interference effect, and fast response time. The obtained results for stability testing are presented in Figure 7. For the repeatability study, ten successive measurements (current density) at 0.83 V were taken (Figure 7a). The relative standard deviation in the measurements was 2.2%, which suggests high stability. Current density reproducibility was also carried out by recording the current density produced by PANI-MnBaO_2_ modified GCE over the period of 30 days (Figure 7b). It was observed that only 4% of the current density decayed after 30 days. The relative standard deviation of the recorded current density response among the GCEs was 5.2%. The obtained results indicate that PANI-MnBaO_2_ is a highly stable and effective sensing material for glucose.

The effect of likely interferents was assessed and the result is presented in Figure 7c. From the result, it could be observed that upon the addition of 10 mM of fructose, ascorbic acid, uric acid, dopamine, and Zn (II) ion. The oxidation peak was unaffected in terms of strength, but little potential drift upon the addition of dopamine. The obtained oxidation peak current upon addition of the listed interferents has a relative standard deviation of 4.8%. Since this value is less than 5%, it could be well assumed that the listed interferents did not interfere with the current density of PANI-MnBaO_2_ in the glucose solution.

Moreover, the response time of the PANI-MnBaO_2_ modified GCE towards oxidation of glucose was examined using the I-V method. The prepared PANI-MnBaO_2_ showed increasing oxidation as early as 2 s and became steady at <10 s (Figure 7d). The fast response time of PANI-MnBaO_2_ modified GCE is extremely important in the real application of the prepared electrode for real-life glucose detection as a point of care device.

#### 3.7.3. Real Sample Analysis

For real sample analysis, human serum (procured from Sigma Aldrich) was used for the study. The obtained result is summarized in Table 1.

The percentage recovery of spiked glucose concentration ranged from 76–110%. The obtained result supports good potential use in real determination of glucose in human systems.

### 3.8. Comparison with Earlier Reported Glucose Electrochemical Sensor

It is imperative to state that most of the reported glucose electrochemical sensing is based on enzyme-aided processes. Due to the limitations of enzymatic-based detection such as high cost, unstable performance, and complicated procedures, a non-enzymatic procedure such as the one used in the current study is preferred [16]. Therefore, this study reports a non-enzymatic method, which is easier, cost-effective, straight forward with better analytical performance. As shown in Table 2, the as-prepared PANI-MnBaO_2_ compared very well with the literature, and even out-performed others. The observed improved glucose sensing performance by PANI-MNBaO_2_ could be ascribed to the combination of unique electrical and catalytic properties of PANI, manganese oxide, and barium oxide. Therefore, this developed approach is a promising analytical technique for glucose determination.

## 4. Conclusions

This study has presented a new material (PANI-MnBaO_2_ composite) for application in glucose electrochemical sensing. The prepared PANI-MnBaO_2_ was characterized morphologically, optically, and electrochemically. PANI-MnBaO_2_ exhibited good performance in glucose electrochemical sensing in terms of sensitivity, response reproducibility, response repeatability, and fast response time. Therefore, the results obtained in this study reveal that PANI-MnBaO_2_ nanocomposite is a promising electrode material for sensitive detection of glucose in the human system for human health monitoring and protection.

## Figures and Tables

**Table 1:** Result of real sample analysis.

Sample	Replicate Number	Spiked Concentration (mM)	Detected Concentration (mM)	Bias	RSD (%)	Recovery (%)
Human serum Albumin	3	0.5	0.38 ± 0.02	−0.12	5.2	76
	3	1	0.85 ± 0.019	−0.15	2.2	85
	3	2	2.2 ± 0.07	0.2	3.18	110

**Table 2:** Reported current density of the bioanode in the literature.

Electrode Materials	LR (mM)	FDR (mM)	LOD (µM)	Sensitivity (µAmM^−1^ cm^−2^)	Ref
PANI/GO/CuO	0–13	0–20	1.5	1252	[33]
PDDA-graphene/CuO	0.04–4	0.04–4	0.2	4982.2	[34]
Au NPs/PANI	0.01–10	0.01–10	3.05	150	[35]
Graphene/polyaniline-co-diphenylamine	0.001–1	0.001–1	0.1	500	[36]
CuO/NiO/PANI	0.02–2.5	0.02–2.5	2.0	-	[37]
PANI/CuNi	1–7	1–10	0.2	1030	[38]
Ni2(dihydroxyterephtalic acid) MOFs	0.04–0.8	0.04–6	1.46	40.95	[39]
Polypyrrole-MOFs	0.02–0.5	0.02–3	1.13	1805	[40]
MXene-Cu_2_O	0.01–30	-	2.83	11.061	[41]
**PANi/MnBaO_2_/GCE**	**0.05–1.6**	**0.05–10**	**0.06**	**128**	**This Study**

## Data Availability

The authors of this article confirm that all the associated data related to this article are included in this manuscript and its Supplementary Materials. Raw data supporting this article is available from the corresponding author upon request.

## Supplementary Materials

The following are available online at https://www.mdpi.com/article/10.3390/polym14153047/s1, Figure S1: The equivalent circuit diagram, Table S1: Values of electrochemical circuit parameters for bare GE, Table S2: Values of electrochemical circuit parameters for PANI-MnBaO2 modified GCE.
