# Supplementary material for: Sensitive Non-Enzymatic Glucose Electrochemical Sensor Based on Electrochemically Synthesized PANI/Bimetallic Oxide Composite
Source: Polymers (Basel). 2022 Jul 27;14(15):3047. doi: 10.3390/polym14153047 (PMC9370187; doi:10.3390/polym14153047)
Supplement: Supplementary file 1 [file polymers-14-03047-s001.zip › polymers-1780479-supplementary.doc-7.27.pdf]

## Electronic Supporting Information

# Sensitive non-enzymatic glucose electrochemical sensor based on electrochemically synthesized PANI/Bimetallic oxide composite

***Dr Anish Khan*

### S1: Electrochemical Impedance Spectroscopy (EIS) study

The circuit diagram for the Nyquist plot is given below (Fig. S1); where Rs denotes solution resistance, Rct denotes charge transfer resistance, CPE denotes constant phase element, and C denotes capacitance.

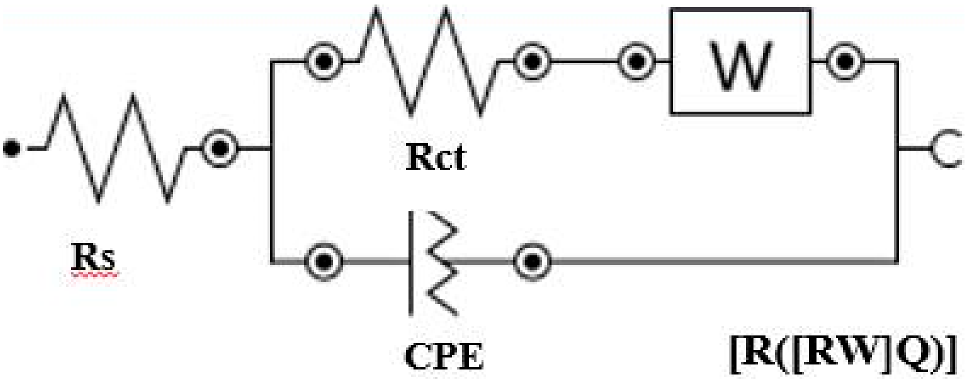


Figure S1: The equivalent circuit diagram

Table S1: Values of electrochemical circuit parameters for bare GE

| Element | Parameter | Value |
|---|---|---|
| Rs (Ω) | R | 120 |
| Rct (Ω) | R | 1470 |
| CPE (μΩ) | Y0 | 2.68 |
| | N | 0.99721 |

Table S2 Values of electrochemical circuit parameters for PANI-$MnBaO_2$ modified GCE

| Element | Parameter | Value |
|---|---|---|
| Rs (Ω) | R | 197 |
| Rct (Ω) | R | 198 |
| CPE (μΩ) | Y0 | 10.92 |
| | N | 0.99721 |
